# Pharmacological Inhibition of O-GlcNAc Transferase Promotes mTOR-Dependent Autophagy in Rat Cortical Neurons

**DOI:** 10.3390/brainsci10120958

**Published:** 2020-12-09

**Authors:** Md. Ataur Rahman, Yoonjeong Cho, Hongik Hwang, Hyewhon Rhim

**Affiliations:** 1 Center for Neuroscience, Korea Institute of Science and Technology (KIST), Seoul 02792, Korea; ataur1981rahman@hotmail.com (M.A.R.); h14009@kist.re.kr (Y.C.); hongik.kist@gmail.com (H.H.); 2 Division of Bio-Medical Science and Technology, KIST School, Korea University of Science and Technology (UST), Seoul 02792, Korea

**Keywords:** cortical neuron, O-GlcNAcylation, O-GlcNAc transferase (OGT), autophagy, mTOR, LC3 puncta

## Abstract

O-GlcNAc transferase (OGT) is a ubiquitous enzyme that regulates the addition of β-N-acetylglucosamine (O-GlcNAc) to serine and threonine residues of target proteins. Autophagy is a cellular process of self-digestion, in which cytoplasmic resources, such as aggregate proteins, toxic compounds, damaged organelles, mitochondria, and lipid molecules, are degraded and recycled. Here, we examined how three different OGT inhibitors, alloxan, BXZ2, and OSMI-1, modulate O-GlcNAcylation in rat cortical neurons, and their autophagic effects were determined by immunoblot and immunofluorescence assays. We found that the treatment of cortical neurons with an OGT inhibitor decreased O-GlcNAcylation levels and increased LC3-II expression. Interestingly, the pre-treatment with rapamycin, an mTOR inhibitor, further increased the expression levels of LC3-II induced by OGT inhibition, implicating the involvement of mTOR signaling in O-GlcNAcylation-dependent autophagy. In contrast, OGT inhibitor-mediated autophagy was significantly attenuated by 3-methyladenine (3-MA), a blocker of autophagosome formation. However, when pre-treated with chloroquine (CQ), a lysosomotropic agent and a late-stage autophagy inhibitor, OGT inhibitors significantly increased LC3-II levels along with LC3 puncta formation, indicating the stimulation of autophagic flux. Lastly, we found that OGT inhibitors significantly decreased the levels of the autophagy substrate p62/SQSTM1 while increasing the expression of lysosome-associated membrane protein 1 (LAMP1). Together, our study reveals that the modulation of O-GlcNAcylation by OGT inhibition regulates mTOR-dependent autophagy in rat cortical neurons.

## 1. Introduction

The cycling of O-GlcNAcylation post-translational modification on the hydroxyl group of serine or threonine residues, known as O-GlcNAcylation, is catalyzed by O-GlcNAc transferase (OGT) and O-GlcNAcase (OGA) [1,2,3], which regulates essential cellular processes, including metabolism, transcription, protein–protein interaction, proteostasis, stress response, protein stability, subcellular localization, and autophagy [4,5,6,7]. Although OGT is expressed ubiquitously, its expression is particularly enriched in neurons, mainly in the nucleus as well as in synapses [8], implicating the role of OGT in regulating neuronal processes. Indeed, previous studies demonstrated that O-GlcNAcylation modulates the induction of long-term depression through the AMPA receptor subunit GluA2 as well as activity-dependent transcription through the cAMP-responsive element binding protein [4]. Moreover, it has been suggested that O-GlcNAcylation modification, mediated by OGT, exerts a neuroprotective function in neurodegenerative diseases by interfering with the production of toxic aggregates formed with α-synuclein, tau, amyloid precursor protein (APP) or neurofilament M (NFM) [9,10,11], and the cycling of O-GlcNAcylation was shown to modulate neurodegenerative phenotypes in *Caenorhabditis elegans*, such as amyloid β-peptide, polyglutamine, and tauopathy expansion. However, it remains to be examined whether O-GlcNAcylation directly contributes to the neuroprotective function by inducing autophagic activity in neurons, and the mechanisms by which O-GlcNAcylation regulates autophagic activity remain unknown at the molecular level. Therefore, revealing the association between autophagy and the regulation of O-GlcNAcylation in neuronal cells may provide essential implications for the treatment of neurodegenerative diseases.

A previous study reported that the cortical brain tissue of human patients with Alzheimer’s disease (AD) has considerably decreased OGT levels [1], implying that O-GlcNAcylation modification plays a critical role in the maintenance of neuronal health, and that dysfunction of O-GlcNAcylation likely contributes to the pathophysiology of neurodegenerative disorders [1]. The essential role of OGT and O-GlcNAcylation in brain functions is further supported by the findings that a decrease in O-GlcNAcylation levels accompanies the neuronal and cognitive hallmarks of brain aging in the hippocampus, and impairs neuronal plasticity and cognition function in aged mouse brain [12]. Interestingly, the stimulation of autophagy via mTOR-mediated inhibition was shown to reduce OGT activity, leading to decreased O-GlcNAcylation levels in human HepG2 cells [13]. Moreover, it has been found that OGT inhibition by alloxan or OGT siRNA up-regulated LC3 puncta formation in cortical astrocytes, and the simultaneous treatment of chloroquine (CQ), an autophagy inhibitor, cooperatively elevated autophagy while diminishing total O-GlcNAcylation levels [14]. However, while mTOR was shown to play an important role in regulating autophagy in mouse cortical astrocytes and neuroblastoma cells [15,16,17,18,19], the precise mechanism that synchronizes autophagy with O-GlcNAcylation levels in neuronal cells is not fully understood.

Neurons are the fundamental units of the nervous system and brain, which play an important role in cellular dynamic developments for their appropriate function—for example, protein synthesis, neuronal growth, axonal migration, synapse formation and elimination, as well as maturation and degradation [20]. Besides, autophagic function of neurons are required as a housekeeper to conserve cellular homeostasis—for example, to maintain organelle or protein quality control. In contrast, neuronal autophagy might be used as a fighter against several disease states, for instance, increasing the accumulation of aggregated protein as well as damaged or injured organelles to inhibit cell death [21]. It has been found that excessive or defective autophagic flux could contribute to neuronal cell death and neurodegenerative disorders. Therefore, neuronal protein quality controls are directly associated with neuronal pathology and physiology. Autophagy, a cellular mechanism to degrade aggregated and dysfunctional components via lysosomes, plays an essential role in maintaining cellular functions and homeostasis [22]. For the initiation of autophagy, membrane nucleation and phagophore formation are required, and pro-LC3 is converted to LC3-I, which consecutively binds to phosphatidylethanolamine (PE) to form LC3-II, eventually promoting phagophore elongation and autophagosome formation. Following the fusion of autophagosomes and lysosomes, termed as an autolysosome, intra-vacuolar LC3-II is degraded. Therefore, monitoring LC3-II formation and degradation serves as a reliable approach to understand the overall autophagy process [23]. In this study, we considered LC3-II as an autophagy marker that is highly expressed in the brain tissue [24]. Although O-GlcNAcylation as well as autophagy are tightly controlled by stress response and nutrient signaling, only few studies have explored the role of O-GlcNAcylation in controlling autophagy. It has been reported that the modulation of O-GlcNAcylation levels by azaserine or glucosamine or by overexpression of OGT or OGA plays an important role in regulating the toxicity of mutant huntingtin in the cellular model of tauopathy and *Drosophila* [25,26]. A decrease in O-GlcNAcylation levels elevates autophagic flux by promoting the fusion of autophagosomes and lysosomes [6,25]. In the present study, we utilized small molecule inhibitors of OGT, alloxan [27,28], BZX2 [29], and OSMI-1 [30], and determined whether and how a decrease in O-GlcNAcylation contributes to autophagy in neurons. Collectively, our findings suggest that the inhibition of O-GlcNAcylation modification stimulates LC3-II expression and suppresses the autophagic substrate p62/SQSTM1 through the mTOR-dependent pathway, thereby revealing an important association between autophagy and O-GlcNAcylation in cortical neurons.

## 2. Materials and Methods

All procedures and protocols concerning the use of animals in this study were reviewed and approved by the Institutional Animal Care and Use Committee of the Korea Institute of Science and Technology (KIST) (Approval No. KIST-2020-106).

### 2.1. Reagents

BXZ2, 3-methyladenine (3-MA), rapamycin, chloroquine diphosphate salt (CQ), alloxan monohydrate, and OSMI-1 were purchased from Sigma-Aldrich (St. Louis, MO 63178, USA). Anti-LC3 (D3U4C) XP^®^ Rabbit mAb (Alexa Fluor^®^ 488 Conjugate), anti-SQSTM1/p62, anti-phospho-S6, anti-total S6, anti-LAMP1, and anti-LC3 antibodies were purchased from Cell Signaling Technology (Danvers, MA 01923, USA). O-linked-N-acetylglucosamine antibody (RL2) was purchased from Thermo Fisher (Rockford, IL 61101, USA). Anti-MAP2 antibody (ab5392) was obtained from Abcam (Cambridge CB2 0AX, UK). Anti-rabbit HRP-linked secondary antibody (#7074P2) and goat anti-mouse HRP-linked secondary antibody (#62-6520) were purchased from Sigma-Aldrich and Invitrogen, respectively. Alloxan, CQ, BXZ2, rapamycin, OSMI-1 were dissolved in DMSO. Then, 3-MA was dissolved in DDW.

### 2.2. Cortical Neuron Cultures

Cortical neurons were obtained from 18-day-old fetal Sprague–Dawley rats with the technique modified from Ryo et al. [31]. Isolated cortex was incubated in a 0.25% pre-warmed trypsin solution at 37 °C for 25 min and the cells were inverted at 5 min intervals. After centrifugation, the cells were mechanically separated using fire-polished Pasteur pipettes and triturated, and then plated in a 12-well and 6-well dish coated with poly-D-lysine. Cells were cultured in a neurobasal medium (#21103-049) containing 2% B-27 supplement, glucose 4500 mg/L, 5% fetal bovine serum (FBS), 2 mM glutamine, 100 U/mL penicillin, and 100 μg/mL streptomycin in a humidified atmosphere of 95% air and 5% CO_2_ at 37 °C. Subsequently, the media were changed to the same composition without FBS twice a week and treated with fluorodeoxyuridine (10 μM) after 7 days in vitro. All experiments were carried out using cortical neurons after 14 days. All techniques and protocols used in this study were approved by the Institutional Animal Care and Use Committee of the Korea Institute of Science and Technology (KIST) (Approval No. KIST-2020-106).

### 2.3. Immunocytochemical Analysis

After treatment, cells were washed with phosphate-buffered saline (PBS) solution and fixed with 100% methanol at −20 °C for 15–20 min. After fixation was completed, cells were washed 3 times with PBS. Block solution was applied for 1 h with 5% normal goat serum containing 0.3% Triton™ X-100 in PBS. After blocking, cells were treated with Alexa Fluor^®^ 488 conjugate anti-LC3-II (1:50) and neuronal marker MAP2 (1:2000) in 1% bovine serum albumin (BSA), 0.3% Triton™ X100, PBS overnight at 4 °C. The next day, the nuclear marker 4′,6-diamidino-2-phenylindole (DAPI) was applied in PBS for 10 to 15 min. After mounting and drying, LC3-II puncta were imaged by confocal microscopy using a Leica Application Suite X (LAS X) microscope (Leica Microsystems, 35578 Wetzlar, Germany).

### 2.4. Immunoblot Analysis

For immunoblotting analysis, neuronal cells grown in a 6-well dish were used. When the cells were properly grown, the drug treatment was performed. After 24 h, the cells were harvested using a radioimmunoprecipitation assay (RIPA) buffer (ELPIS-BIOTECH. Inc., Daejeon 12312, Korea). The collected cell lysates were kept on ice for 30 min. Lysates were centrifuged at 14,500 rpm for 10 min at 4 °C. The upper layer of proteins was separated from the tube. Protein quantification was performed using Bradford (Coomassie) protein assay kits (GenDEPOT, Katy, TX 77494, USA). To separate proteins based on the molecular weight, 8–15% reducing gels were made depending on the protein size. Equivalent quantities of protein samples were loaded in every well and separated by SDS-PAGE. After separation of the proteins, proteins were transferred to a polyvinylidene fluoride (PVDF) membrane. Subsequently, the membrane was blocked with 5% skim milk or BSA for 1 h at room temperature. After being blocked, the membrane was washed with phosphate buffered saline with 0.1% Tween^®^ 20 (PBST) and incubated with a specific primary antibody at 4 °C overnight. The membrane was then washed 3 times with PBST. Then, 5% skim milk or BSA was used to dissolve the secondary antibody conjugated with horseradish peroxide, and the membrane was incubated for a minimum of 2 h at room temperature. Lastly, the membrane was washed three times with PBST and the bands were detected with enhanced chemiluminescence (ECL) kits using the AlphaEase program (Alpha Innotech, San Leandro, CA 94577, USA). In all experiments, the band intensities were quantified using the Image J software.

### 2.5. Autophagic Flux Counting

Puncta formation were counted and analyzed from confocal image of immunocytochemistry analysis which was well described by Rahman et al. [14]. At least 5 cells were counted from each image per condition and average number was plotted in a bar graph and results were presented via standard mean of error (±SEM). Measurements of autophagic flux in vitro were performed using the autophagosomal or lysosomal fusion inhibitors chloroquine (CQ) with 10 µM for 2 h before harvesting or fixing the cells and then probing with autophagosomal marker anti-LC3-II conjugate Alexa Fluor^®^ 488 via immunocytochemistry assay.

### 2.6. Dot Blot Analysis

To perform dot-blot analysis, 1 μL volume of each sample containing 1 μg of proteins was spotted onto a polyvinylidene fluoride (PVDF) membrane. The membrane was dried in air for at least 30 min. After drying, the membrane was blocked in 5% BSA in 1X TBST for 1 h. After blocking, the spotted membrane was incubated with ant-O-GlcNAcylation antibody overnight at 4 °C. The next day, the membrane was washed and subsequently incubated with appropriated secondary antibody conjugate with horseradish peroxidase at room temperature for 1 h in gentle shaking. Having been washed three times with 1X TBST, the signal was detected using an enhanced chemiluminescence (ECL) kits (iNtron Biotechnology, Seoul 462-120, Korea). Protein bands were detected by the AlphaEase program. The band intensities were quantified with ImageJ software’s version JAVAFX.

### 2.7. Statistical Analysis

Two-group comparisons were performed using unpaired Student’s *t*-tests. Multiple comparisons were performed using one-way analysis of variance (ANOVA) followed by Tukey’s multiple comparisons post hoc test. GraphPad Prism 7 software (GraphPad Software Inc., La Jolla, San Jose, CA 95124, USA) was used for all data analysis. Data are presented as the mean ± SEM. Results were considered statistically significant at * *p* < 0.05, ** *p* < 0.01, *** *p* < 0.001, and **** *p* < 0.0001.

## 3. Results

### 3.1. Inhibition of O-GlcNAc Transferase (OGT) Stimulates Autophagy in Rat Cortical Neurons and Neuronal Cell Lines

**Figure 1:**
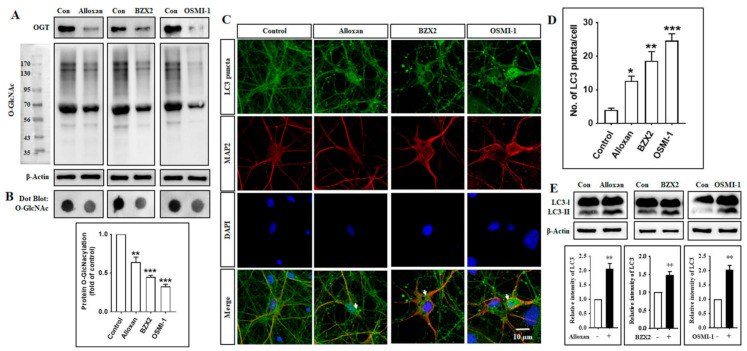
Pharmacological inhibition of O-GlcNAc transferase (OGT) decreases O-GlcNAcylation levels and promotes autophagy in rat cortical neurons. (**A**) Immunoblot analysis of OGT and O-GlcNAcylation levels were determined after treatment of alloxan (5 mM), BZX2 (100 µM), and OSMI-1 (50 µM) for 24 h in rat cortical neurons. (**B**) Dot blot analysis of O-GlcNAcylation levels after alloxan, BZX2, and OSMI-1 treatment for 24 h. Densitometry quantification of O-GlcNAcylation protein was performed by Image J. All statistical analysis was obtained as ±SEM (*n* = 3, ** *p* < 0.01, *** *p* < 0.001). (**C**) Cortical neurons were treated with alloxan, BZX2, and OSMI-1 for 24 h, and the formation of LC3-II puncta was visualized by confocal microscopy following the immunofluorescence staining with anti-LC3 (green) and anti-MAP2, a neuro-specific marker, (red) antibodies. (**D**) LC3-II puncta formation was measured from at least 3 independent random areas from each slide as well as minimum 5 cells which contain puncta was counted and calculated (ns = non-significant, * *p* < 0.05, ** *p* < 0.01, *** *p* < 0.001). (**E**) Immunoblot analysis of LC3 expression levels in rat cortical neurons treated with alloxan, BZX2 or OSMI-1. Densitometry quantification of LC3 levels was performed using Image J. All data are presented as mean ± SEM (*n* = 3, ** *p* < 0.01).

To elucidate how O-GlcNAcylation modification affects autophagy in cortical neurons, we investigated the role of different OGT inhibitors in regulating the autophagic pathway. Toward this aim, cortical neurons were treated with an OGT inhibitor (alloxan, BZX2 and OSMI-1), and changes in O-GlcNAcylation levels and autophagic activities were examined by immunoblot and immunocytochemistry assays. Previous studies have shown that the inhibition of OGT enzymatic activity decreases cellular O-GlcNAcylation levels [1] and promotes autophagy [13,14]. In the present study, we found that all these three inhibitors decreased OGT and O-GlcNAcylation levels (Figure 1A), and additionally we also examined O-GlcNAcylation levels were significantly decreased by these inhibitors determined by dot blot analysis in cortical neurons (Figure 1B). We also found that the number of LC3-II puncta formed per cell was significantly increased by the treatment with alloxan, BZX2, and OSMI-1, as determined by the immunofluorescence staining with anti-LC3-II antibody through counting puncta number (Figure 1C,D). However, alloxan, BZX2 and OSMI-1 treatments significantly increased LC3-II expression in cortical neurons (Figure 1E). Furthermore, the effects of OGT inhibitors in autophagy were also examined in two different neuronal cell lines, human SH-SY5Y neuroblastoma and U87MG glioblastoma. Consistent with the result from cortical neurons, the treatment with alloxan, BZX2, and OSMI-1 for 24 h also significantly enhanced the formation of LC3 puncta in both SH-SY5Y and U87MG cells (Supplementary Figure S1). We also treated the cells with thiamet-G, O-GlcNAcase (OGA) inhibitor [32], and found that thiamet-G treatment increased O-GlcNAcylation and there was no change in LC3-II protein expression examined via immunoblot analysis (Supplementary Figure S2). Therefore, together these data indicate that decreasing O-GlcNAcylation level stimulates autophagy in cortical neurons.

### 3.2. OGT Inhibitors Induce Autophagy in Cortical Neurons in an mTOR-Dependent Manner

**Figure 2:**
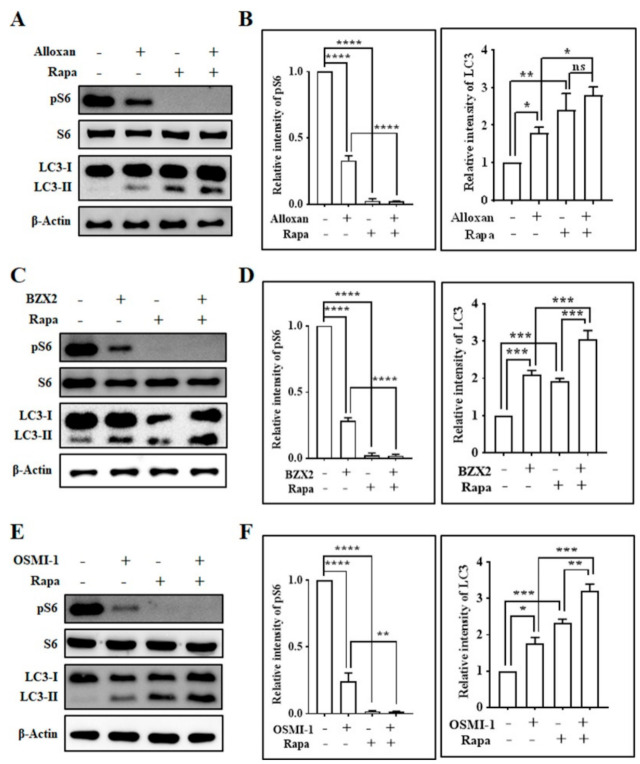
Inhibition of OGT regulates mTOR signaling pathway in rat cortical neurons. (**A**,**C**,**E**) Neurons were pretreated with rapamycin (100 nM) for 30 min before in the absence/presence of alloxan (5 mM), BZX2 (100 µM), and OSMI-1 (50 µM) for 24 h. Cells were harvested and pS6, S6, and LC3 expression levels were examined by immunoblot analysis. (**B**,**D**,**F**) Densitometry analysis of pS6 and LC3 protein levels quantified by Image J. All data are presented as mean ± SEM (*n* = 3, ns; non-significant, * *p* < 0.05, ** *p* < 0.01, *** *p* < 0.001, **** *p* < 0.0001).

Mammalian target of rapamycin (mTOR), a serine/threonine protein kinase, plays an essential role in cell growth, which is composed of two separate cellular complexes: mTOR complex 1 (mTORC1) and mTOR complex 2 (mTORC2) [33]. mTORC1 phosphorylates and activates ribosomal S6 kinase (S6) and regulates protein synthesis [34], and the inhibition of mTORC1 by rapamycin induces autophagy in astrocytes [15,35,36]. Here, we examined whether OGT inhibitor-mediated autophagy induction was regulated by the mTOR-dependent pathway, and found that all OGT inhibitors significantly down-regulated the phosphorylation of S6 (pS6) (Figure 2), although OGT and O-GlcNAcylation levels were decreased by rapamycin and cotreatment with OGT inhibitors (Supplementary Figure S3). Particularly, rapamycin, an mTOR inhibitor, entirely eliminated S6 phosphorylation when applied alone, as well as with OGT inhibitors. Rapamycin also significantly increased the expression of LC3-II protein, and the co-treatment of rapamycin with OGT inhibitors (alloxan, BZX2, OSMI-1) further elevated LC3-II expression levels compared to the case of rapamycin alone in cortical neurons (Figure 2). Together, this result suggests that a decrease in O-GlcNAcylation levels via OGT inhibition reduces mTOR activation and promotes the mTOR-dependent autophagy process in cortical neurons.

### 3.3. Autophagy Inhibitor Blocks OGT Inhibitor-Mediated Autophagy in Cortical Neurons

**Figure 3:**
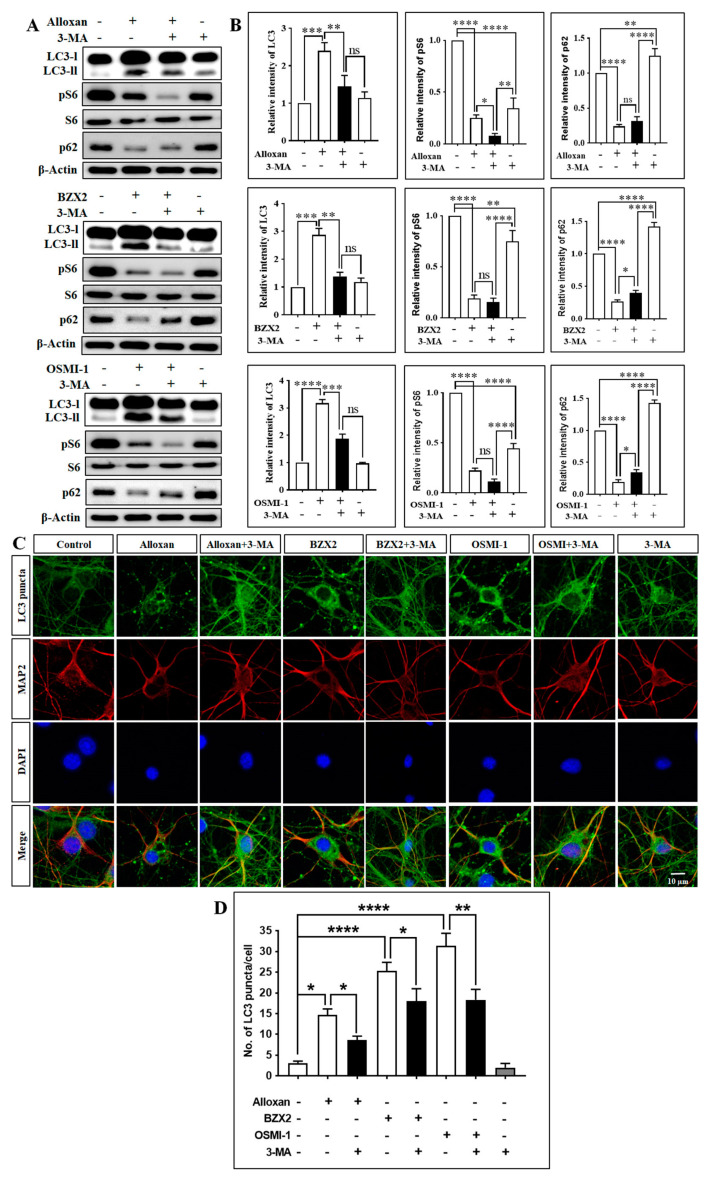
OGT inhibitor-mediated autophagy inhibited by 3-MA in rat cortical neurons. (**A**) Neuronal cells were pre-treated (3 h) with 3-MA (2.5 mM), and subsequently treated with alloxan, BZX2, and OSMI-1 for 24 h. LC3-II/LC3-I, pS6, S6, p62 expressions were determined by immunoblot. (**B**) Band intensities were quantified with Image J, and each protein band intensity was normalized to that of β-actin. All data are presented as mean ± SEM (*n* = 3, ns; non-significant, * *p* < 0.05, ** *p* < 0.01, *** *p* < 0.001, **** *p* < 0.0001). (**C**) Cortical neurons were fixed and stained with Alexa Fluor 488 conjugated-anti-LC3-II antibody (green) and MAP2. Images were acquired with confocal microscopy. (**D**) LC3 puncta formation was measured from at least 3 independent random areas from each slide, as well as a minimum of 5 cells which contained puncta and were counted and calculated (ns = non-significant, * *p* < 0.05, ** *p* < 0.01, **** *p* < 0.0001).

Subsequently, we examined whether the OGT inhibitors-mediated induction of autophagy is modulated by autophagy blocker, 3-methyladenine (3-MA), a well-recognized class III phosphatidylinositol 3 kinase (class III PI3-kinase) inhibitor commonly used in autophagy inhibition experiment [37]. First, we checked the effect of 3-MA on OGT and O-GlcNAcylation levels. Here, we found that 3-MA alone increased OGT and O-GlcNAcylation levels in cortical neuronal cells, although the cotreatment of 3-MA and OGT inhibitors decreased OGT and O-GlcNAcylation expression (Supplementary Figure S4). From our investigations, we found that 3-MA pretreatment significantly decreased the OGT inhibition-mediated LC3-II expression while 3-MA alone did not change LC3-II levels (Figure 3A,B). This indicates that OGT inhibition-mediated autophagy was dependent on the class III PI3-kinase-mediated pathway. Moreover, mTOR downstream protein phospho-S6 was significantly downregulated by OGT inhibitors (Figure 3A,B), and the cotreatment with 3-MA and OGT inhibitors (alloxan, BZX2, and OSMI-1) significantly reduced the expression of phospho-S6 compared to cells treated with 3-MA alone. In addition, the modulation of autophagic flux by O-GlcNAcylation was further verified by examining p62, also known as sequestosome 1 (SQSTM1). p62 is an essential component of autophagic substrates that directly bind to LC3, and is involved in diverse cellular functions [38]. Importantly, p62 functions as a positive regulator of mTORC1 and acts as a scaffold on lysosomal membranes [39]. During the autophagy process, p62 is absorbed into the autophagosome and subsequently degraded by autophagy, thereby limiting mTORC1 activity [40]. Consequently, the inhibition of autophagy may lead to an increase in total p62 levels [41], which can be utilized to monitor autophagic flux. We also found that the inhibition of class III PI3-kinase by 3-MA significantly increased the levels of p62 compared to the control (Figure 3A,B), while the cotreatment of OGT inhibitors and 3-MA significantly reduced the levels of p62 compared with 3-MA alone. In addition, the cotreatment of OGT inhibitors and 3-MA slightly, but significantly, increased p62 expression compared to the treatment with OGT inhibitors alone, except for alloxan (Figure 3A,B). Similarly, immunostaining data showed that 3-MA pre-treatment also significantly prevented the formation of LC3-II puncta-induced by OGT inhibitors determined through counting of puncta number per cells (Figure 3C,D). Therefore, these results suggest that the class III PI3K inhibitor has an essential function in modulating OGT inhibition-mediated O-GlcNAcylation regulation and autophagy induction in cortical neurons.

### 3.4. OGT Inhibitors Induce Autophagic Flux and Degrade the Autophagy Substrate p62/SQSTM1 in Cortical Neurons

**Figure 4:**
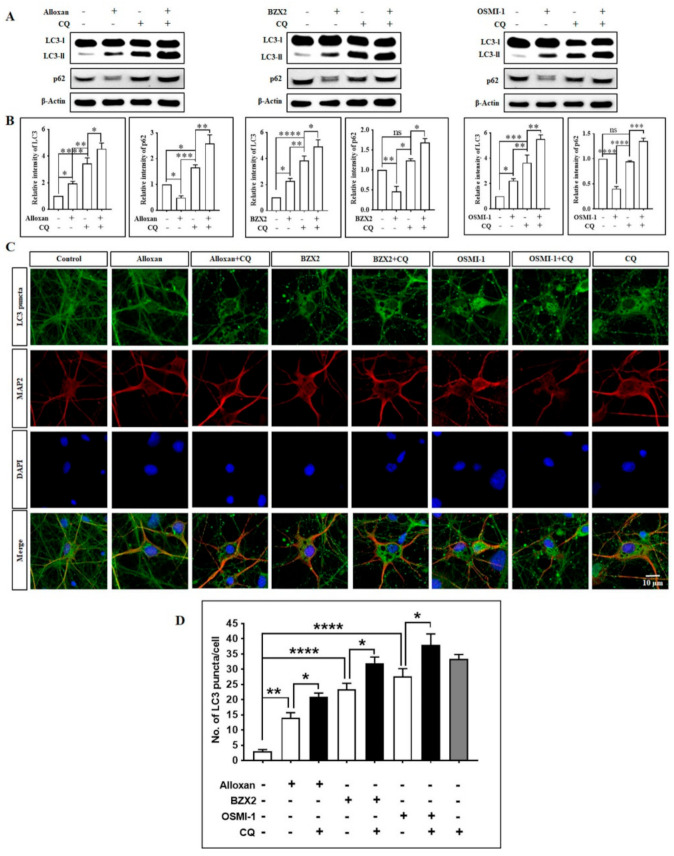
OGT inhibitors stimulate autophagic activities in rat cortical neurons. (**A**) After the treatment with alloxan, BZX2, and OSMI-1, cortical neurons were incubated with chloroquine (10 µM) for 2 h before harvesting. LC3 and p62 expression levels were determined by immunoblot analysis. (**B**) Data are presented as mean ± SEM (*n* = 3, ns; non-significant, * *p* < 0.05, ** *p* < 0.01, *** *p* < 0.001, **** *p* < 0.0001). (**C**) Cortical neurons were treated with alloxan, BZX2, or OSMI-1 for 24 h, and chloroquine was applied for 2 h before fixation. The neurons were then stained with Alexa fluor 488 conjugated-anti LC3-II antibody (green) and MAP2 (red). Images were acquired with confocal microscopy. (**D**) LC3 puncta formation was measured from at least 3 independent random areas from each slide. A minimum of 5 cells were counted and calculated, which contained puncta (ns = non-significant, * *p* < 0.05, ** *p* < 0.01, **** *p* < 0.0001).

Given the observation that OGT inhibitors induce autophagy, we next examined whether alloxan, BZX2, and OSMI-1 regulates autophagic flux in cortical neurons using LC3 turnover assay [23]. In this assay, cells are incubated with a lysosomotropic agent, such as chloroquine (CQ) [42], to prevent the acidification of lysosomes or the fusion between autophagosomes and lysosomes, which results in the blockade of LC3-II degradation and the subsequent accumulation of LC3-II levels [43]. Therefore, the comparison of LC3-II levels following drug treatment in the presence or absence of an autophagy inhibitor essentially determines the quantity of LC3-II provided to lysosomal degradation [14,44]. To elucidate whether alloxan, BZX2, and OSMI-1 controls autophagic flux, LC3 levels were examined in cortical neurons in the presence or absence of CQ, the late-stage autophagy inhibitor. CQ alone significantly up-regulated LC3-II protein expression, and the co-treatment of CQ with OGT inhibitors further increased LC3-II expression compared to CQ alone (Figure 4A,B), which was also confirmed by immunofluorescence analysis (Figure 4C,D), although OGT and O-GlcNAcylation levels were decreased by CQ and OGT inhibitor cotreatment and CQ alone (Supplementary Figure S5).

To study the specific p62 function in OGT inhibitor-associated autophagy, we performed p62 immunoblotting analysis, and found that alloxan, BZX2, and OSMI-1 treatment significantly reduced the levels of p62 expression (Figure 4A,B). By performing the LC3 turnover assay, we also found that autolysosome accumulation was prevented by CQ; as a result, p62 levels were considerably enhanced following the treatment with alloxan, BZX2, and OSMI-1, due to the impaired deprivation of p62 (Figure 4A,B). Combined together, these results demonstrate that the reduction in O-GlcNAcylation by OGT inhibitors considerably increases autophagic flux in cortical neurons.

### 3.5. OGT Inhibitors Increase the Expression of Lysosome-Associated Membrane Protein 1 (LAMP1) in Cortical Neurons

**Figure 5:**
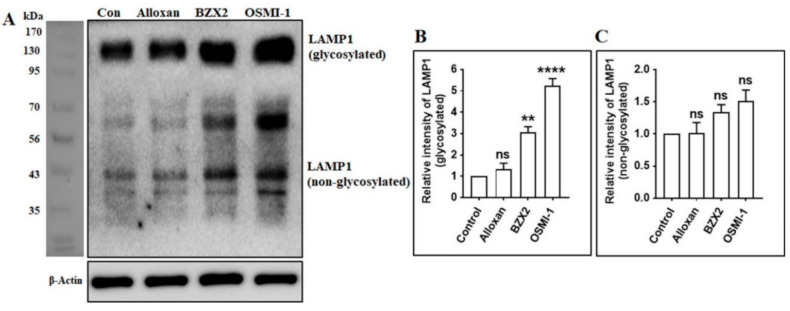
OGT inhibition stimulates the fusion of autophagosomes and lysosomes by inducing LAMP1 expression. (**A**) Cortical neurons were treated with alloxan, BZX2, and OSMI-1 for 24 h. The expression levels of LAMP1 were determined by immunoblot analysis. (**B**,**C**) Glycosylated and non-glycosylated LAMP1 was determined by densitometry analysis via Image J and presented as mean ± SEM (*n* = 3, ** *p* < 0.01, **** *p* < 0.0001, ns = non-significant).

Autolysosomes are generated by the fusion of autophagosomes and lysosomes, which requires a combination of lysosomal proteins, such as Lysosomal-associated membrane protein 1 (LAMP1) [45,46]. LAMP1, as well as LAMP2, are generally found in lysosomes in addition to late endosomes [47]. LAMP1 and LAMP2 represent about 50% of lysosomal membrane proteins. They have a large number of N-glycans, as well as O-glycans, with a molecular mass ranging from 42 kDa (non-glycosylated) up to between 90 and 120 kDa for the heavily glycosylated forms [48] Figure 5A). To examine whether the generation of autolysosomes is regulated by O-GlcNAcylation, the expression levels of glycosylated and non-glycosylated LAMP1 were determined by immunoblotting following the inhibition of OGT. We found that LAMP1 glycosylated protein expressions were significantly (except alloxan) increased after the treatment with OGT inhibitors in cortical neurons (Figure 5A,B), although non-glycosylated LAMP1 was non significantly increased by OGT inhibition (Figure 5A,C), indicating that the modulation of O-GlcNAcylation activates autophagy by promoting the fusion of autophagosomes with lysosomes.

## 4. Discussion

Several studies have proposed that the suppression of O-GlcNAcylation modification improves autophagy [6,22]. For example, a decrease in O-GlcNAcylation levels through OGT knockdown improves autophagy in animal cells and *C. elegans* [6]. However, the mechanism of O-GlcNAcylation-mediated autophagy is not fully understood at the molecular level, and it remains unclear how OGT inhibition regulates autophagy signaling. To understand the process of O-GlcNAcylation-dependent modulation of autophagy, we utilized rat cortical neurons as an in vitro culture model in this study. Herein, our data showed that an OGT inhibitor (alloxan, BZX2, or OSMI-1) modulates O-GlcNAcylation levels and promotes autophagy in neuronal cells.

Due to the significant implication of OGT in regulating cellular processes, numerous small molecules have been described to inhibit OGT activity in both in vitro and in vivo experiments, including APBT, 1-(4-acetamidophenyl)-4-([1,1-biphenyl]-4-yl)-1H-1,2,3-triazole, APNT, 1-(4-acetamidophenyl)-4-(naphthalen-2-yl)-1H-1,2,3-triazole [49], BADGP, benzyl-2-acetamido-2-deoxy-α-D-galactopyranoside (derivative of N-acetylgalactosamine) [50], Ac_4_-5SGlcNAc (metabolic precursor) [51], alloxan (uracil mimic) [49], benzoxazolinone (BZX2) (neutral diphosphate mimic derivative) [52], and OSMI-1 (derivative of quinolinone-6-sulfonamide) [30]. Alloxan directly inhibits OGT in a concentration-dependent manner with an IC_50_ value of 9 mM [53], similar to that of streptozotocin which exhibits significant inhibition at the concentration of 5 mM [54]. It has been reported that some substrates and bisubstrates mimic OGT inhibition in vitro; however, these inhibitors are not membrane permeable and hence are ineffective in cells [55,56]. Alloxan has been found to reduce dialuric acid wherever it produces free radicals—for example, H_2_O_2_—and these free radicals could damage the intracellular components which destruct pancreatic β-cells, consequently causing insulin-dependent Diabetes mellitus [57]. Alloxan furthermore selectively prevents glucose-mediated secretion of insulin via its capacity to prevent the β-cell glucose sensor glucokinase in streptozotocin-induced diabetes [58]. Benzoxazolinone (BZX2), another small molecule that comprises a five-heteroatom dicarbamate core with an IC_50_ value of 11.9 μM [49], deactivates OGT by creating a carbonyl crosslink in the OGT active site, which significantly decreases O-GlcNAcylation levels at the concentrations of 100 μM [59]. BZX2 treatment enhanced phosphorylation of tau at Ser199 and Ser396, which had been considerably decreased through the Thiamet G treatment, and repressed tau aggregation [29]. OGT inhibition by BZX2 initiated the reverse change, which indicated the defensive character of O-GlcNAcylation modification in the pathology of tau [2]. Additionally, we also studied OSMI-1, a small molecule OGT inhibitor, established through a high-throughput screening hit with 50 μM concentration [30]. An important aspect of OSMI-1 is that it is cell-permeable and prevents O-GlcNAcylation of proteins in numerous mammalian cell lines. OSMI-1 inhibits human OGT with an IC_50_ value of 2.7 μM, as determined by a radiometric capture assay [30]. These OGT inhibitors serve as an important tool to modulate O-GlcNAcylation levels and to influence autophagy in neuronal cells.

Our previous studies have demonstrated that the modulation of O-GlcNAcylation levels regulates neuronal properties [2] and the OGT inhibitors described above effectively suppress O-GlcNAcylation levels in brain cells [29,60]. During starvation conditions, it has also been found that perturbations in O-GlcNAcylation cycling lead to elevated LC3 protein expression in *C. elegans* [61,62]. Additionally, a recent study has shown that O-GlcNAcylation also hinders autophagy by suppressing Atg protein levels and autophagosome formation in the *Drosophila melanogaster* model [63]. In the present study, we found that a decrease in O-GlcNAcylation levels mediated by OGT inhibitors considerably increases autophagy in cortical neurons and neuronal cell lines (Figure 1), suggesting that the modulation of O-GlcNAcylation cycling serves as an important mechanism in controlling autophagy in neuronal cells.

mTOR signaling plays a significant role in autophagy induction and termination [35], and the inhibition of mTOR pathway by rapamycin activates autophagy [15]. It has been found that rapamycin decreased basal O-GlcNAcylation levels, although rapamycin incompletely rescued hyper-O-GlcNAcylation-mediated autophagic inhibition in neurons treated by thiamet G [64]. Interestingly, another study has shown that rapamycin reduces OGT expression and O-GlcNAcylation levels in HepG2 cells, which contributes to the modulation of OGT stability by mTOR as well as the initiation of autophagy [64]. In the current study, we found that the pharmacological inhibition of OGT considerably down-regulates mTOR pathway and stimulates autophagy. In addition, we also demonstrated that the co-treatment of rapamycin with OGT inhibitor (alloxan, BZX2, OSMI-1) further enhanced the levels of LC3-II compared to rapamycin alone (Figure 2), highlighting that the inhibition of OGT and mTOR cooperatively modulates the induction of autophagy in cortical neurons.

In autophagy studies, it is important to determine whether the accumulation of autophagosome is due to the promotion of autophagy or the inhibition of downstream autophagy processes [65]. In this regard, measuring the total amounts of autophagosomes as well as autolysosome recognition helps to differentiate the two possible scenarios concerned with overall autophagic flux. The quantification of LC3-II expression levels by immunoblotting is one of the most effective approaches to reliably detect the assembly and the degradation of autophagosomes in the cytosol [65]. In the present study, we found that the variance in LC3-II levels in the absence and presence of CQ treatment was greater in response to the treatment with an OGT inhibitor, indicating that a decrease in O-GlcNAcylation levels increased autophagic flux activity (Figure 4). These findings are consistent with our previous study, in which OGT inactivation in mouse primary cortical astrocytes enhances autophagic flux in response to the reduction in O-GlcNAcylation levels [14]. Moreover, the quantification of the autophagic substrate p62/SQSTM1 serves as another effective approach to determine autophagic flux. Although p62 and LC3 are transcriptionally regulated by autophagy, the clarification of these two proteins is a key indicator of autophagic flux [66]. Thus, we further quantified p62 to evaluate the autophagic flux prompted by OGT inhibitors, and found that the blockade of autophagosome-lysosome fusion through CQ further enhanced p62 levels in OGT inhibitor-treated neuronal cells (Figure 4). This result is in agreement with the previous study in which the autophagic flux is impaired by trimethyltin in astrocytes treated with lysosomotropic agents [67], indicating that the pharmacological inhibition of OGT is capable of increasing the autophagic flux in cortical neurons.

The formation of autophagosomes, which is ultimately fused to lysosomes, is essential for the degradation and recycling of cellular elements during autophagy [68]. A previous study reported that a decrease in O-GlcNAcylation levels increases the accumulation of autophagosomes and lysosomes, and stimulates autophagy, which is correlated to the reduction in mHtt aggregation in fly models [25]. On the other hand, O-GlcNAcylation modification of SNARE proteins, such as SNAP29, prevents autophagic flux as well as autophagosome-lysosome fusion [6]. Nonetheless, the precise relationship between O-GlcNAcylation and the formation of autophagosomes remained unclear. In this study, we revealed that the inhibition of OGT activity significantly enhanced autolysosome formation by activating with glycosylated and non-glycosylated LAMP1 expression (Figure 5). A similar finding was also observed where the knockdown or the inhibition of OGT stimulates the formation and maturation of autophagosome in mouse cortical astrocytes [14], together indicating that a change in O-GlcNAcylation levels modulates the fusion of autophagosome and lysosomes in cortical neurons. Together, this evidence has suggested that O-GlcNAcylation cycling might be a therapeutic target against neurodegenerative diseases.

It has been found that O-GlcNAcylated proteins, OGT and OGA, are highly expressed in the brain, specifically in the cerebellar cortex and hippocampus [69]. Increasing O-GlcNAcylation levels by the OGA inhibitor, NButGTA, was shown to reduce Aβ40, Aβ42 peptides levels and plaque formation, resulting in cognition improvement in the 5xFAD mouse model of Alzheimer’s disease [70]. Another OGA inhibitor, PUGNAc, reduced tau phosphorylation in PC12 cells overexpressing human tau [10], and Thiamet-G, OGA inhibitor, also considerably decreased tau aggregation and prevented neuronal cell loss in vivo in tauopathy JNPL3 mice and 3×Tg-AD mouse model [71]. Therefore, targeting OGA may have significant implications for the treatment of neurodegenerative disorders. In contrast, forebrain-specific loss of OGT was shown to cause hyperphosphorylation of tau, amyloidogenic Aβ-peptides formation, neuroinflammation, and memory impairments in adult mice [1], and shRNA-mediated knockdown of OGT increased phosphorylation of tau in HEK cells in AD model [72]. Besides, the knockdown of OGT or O-GlcNAcylation sites mutation in SNAP29 protein improves the binding of autophagosome-lysosome enhancing autophagic flux in HeLa cells and the *C. elegans* model [6]. In this regard, O-GlcNAcylation levels of neuronal proteins are closely associated with brain functions, and the levels of O-GlcNAcylation should be maintained within adequate ranges to support normal brain functions.

Considering that autophagy is involved in the degradation of abnormal protein aggregates and the clearance of misfolded proteins [6,73,74,75], the stimulation of autophagic activity may serve as an effective approach in treating neurodegenerative disorders [18,76,77]. Therefore, based on the role of OGT inhibitors in facilitating autophagic activity in cortical neurons, we propose the modulation of O-GlcNAcylation levels by OGT inhibition as a potential therapeutic approach to prevent and treat neurodegenerative disorders associated with a deficit in the autophagy process.

## Supplementary Materials

The following are available online at https://www.mdpi.com/2076-3425/10/12/958/s1, Figure S1: Effects of OGT inhibitors on human neuroblastoma and glioblastoma cells; Figure S2: Effects of thiamet-G in rat cortical neurons; Figure S3: Inhibition of OGT regulates mTOR signaling pathway in rat cortical neurons; Figure S4: Effects of CQ on OGT inhibitor stimulated autophagic activities in rat cortical neurons; Figure S5: Effects of CQ on OGT inhibitor stimulated autophagic activities in rat cortical neurons.
