# Supplementary material for: Pharmacological Inhibition of O-GlcNAc Transferase Promotes mTOR-Dependent Autophagy in Rat Cortical Neurons
Source: Brain Sci. 2020 Dec 9;10(12):958. doi: 10.3390/brainsci10120958 (PMC7763293; doi:10.3390/brainsci10120958)
Supplement: Supplementary file 1 [file brainsci-10-00958-s001.pdf]

Supplementary Information

Pharmacological inhibition of O-GlcNAc transferase promotes mTOR-dependent autophagy in rat cortical neurons

Md. Ataur Rahman[1#], Yoonjeong Cho[1, 2#], Hongik Hwang[1], Hyewhon Rhim[1, 2*]

[1]Center for Neuroscience, Korea Institute of Science and Technology (KIST), Seoul, Republic of Korea; [2]Division of Bio-Medical Science and Technology, KIST School, Korea University of Science and Technology (UST), Seoul 02792, Republic of Korea.

Running title: OGT inhibition stimulates autophagy

[#]These authors contributed equally to this work.

***Correspondence should be addressed to:** Hyewhon Rhim, Center for Neuroscience, Brain Science Institute, Korea Institute of Science and Technology (KIST), 5 Hwarang-ro 14-gil, Seongbuk-gu, Seoul 02792, Republic of Korea. Tel.: +82-2-958-5923, Fax: +82-2- 958-6937. E-mail: hrhim@kist.re.kr

Materials and methods

Neuroblastoma and glioblastoma cell culture

Human SH-SY5Y neuroblastoma and U87MG glioblastoma cells were grown at 37 °C in a humidified at 5% CO_2 incubator. DMEM culture medium with 10% FBS was used for cell culture in addition with 100 U/ml penicillin and 100 μg/ml streptomycin were added in the medium.

Immunocytochemistry analysis

After treatment, SH-SY5Y and U87MG cells were washed with 1X ice cold PBS and fixed with methanol (100%) at -20°C at least 15 min. After fixing, washed 3 times through 1X PBS and blocked by 5% normal goat serum compose of 0.3% Triton™ X-100 in 1X PBS at 1 h. Cells were incubated with anti-LC3-II conjugate Alexa Fluor® 488 (1:50) in 1% BSA and 0.3% Triton™ X100 dissolve in 1X PBS overnight at 4°C. DAPI was added in 1X PBS for 10 min during washing time. LC3-II puncta were visualized and captured by confocal microscopy of Leica Application Suite X (LAS X) (Leica Microsystems, Germany). Puncta formation were counted and analyzed from confocal image of immunocytochemistry analysis. At least 5 cells were counted from each image per condition and average number was plotted in a bar graph and results were presented via standard mean of error (±SEM).

Immunoblot analysis

For immunoblotting analysis, neuronal cells grown in a 6-well dish were used. When the cells were properly grown, the drug treatment was performed. After 24 h, the cells were harvested using a radioimmunoprecipitation assay (RIPA) buffer (ELPIS-BIOTECH. Inc., Daejeon, Korea). Equivalent quantities of protein samples were loaded in every well and separated by SDS-PAGE. After separation of the proteins, proteins were transferred to a polyvinylidene fluoride (PVDF) membrane. After being blocked, the membrane was washed with phosphate buffered saline with 0.1% Tween® 20 (PBST) and incubated with a specific primary antibody at 4°C overnight. The membrane was then washed 3 times with PBST. 5% skim milk or BSA was used to dissolve the secondary antibody conjugated with horseradish peroxide, and the membrane was incubated for a minimum of 2 h at room temperature. Lastly, the membrane was washed three times with PBST and the bands were detected with enhanced chemiluminescence (ECL) kits.

Results

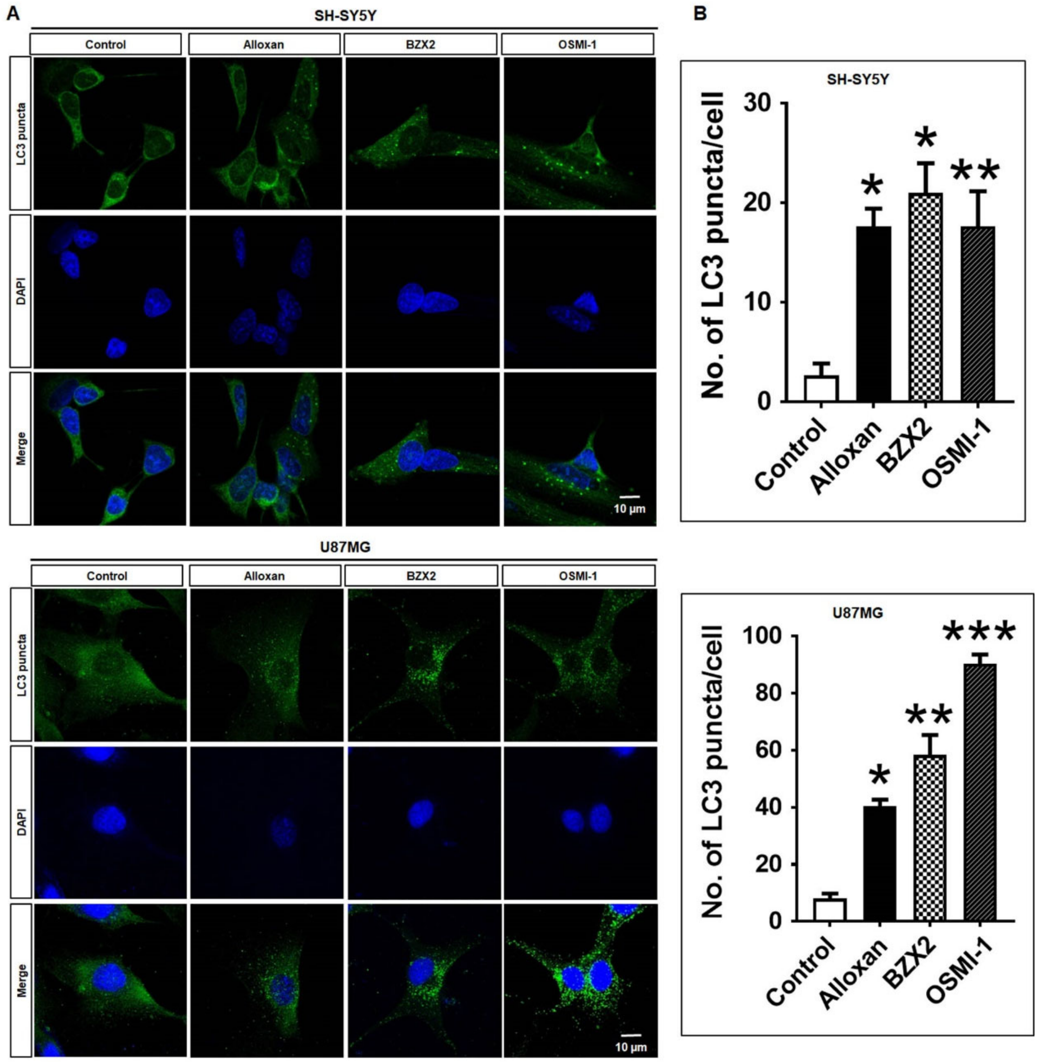

Supplementary Figure 1: Effects of OGT inhibitors on human neuroblastoma and glioblastoma cells. (A) SH-SY5Y and U87MG cells were treated with alloxan, BZX2, and OSMI-1 for 24 h**.** LC3 puncta were determined by immunofluorescence staining with anti-LC3 (green) using a confocal microscopy. (B) Statistical analysis of LC3 puncta were performed by ±SEM (*$p < 0.05$, **$p < 0.01$), ***$p < 0.001$).

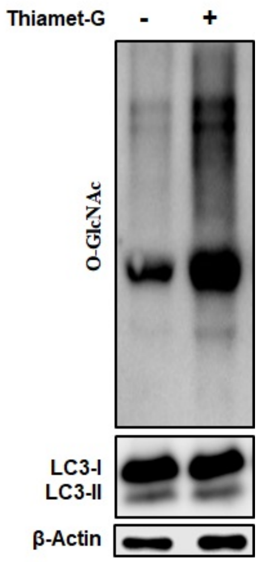

Supplementary Figure 2: Effects of thiamet-G in rat cortical neurons. Representative O-GlcNAc and LC3 expressions were determined by immunoblot via treatment of thiamet-G (1 µM) for 24 h in rat cortical neuronal cells. B-actin was used as a loading control.

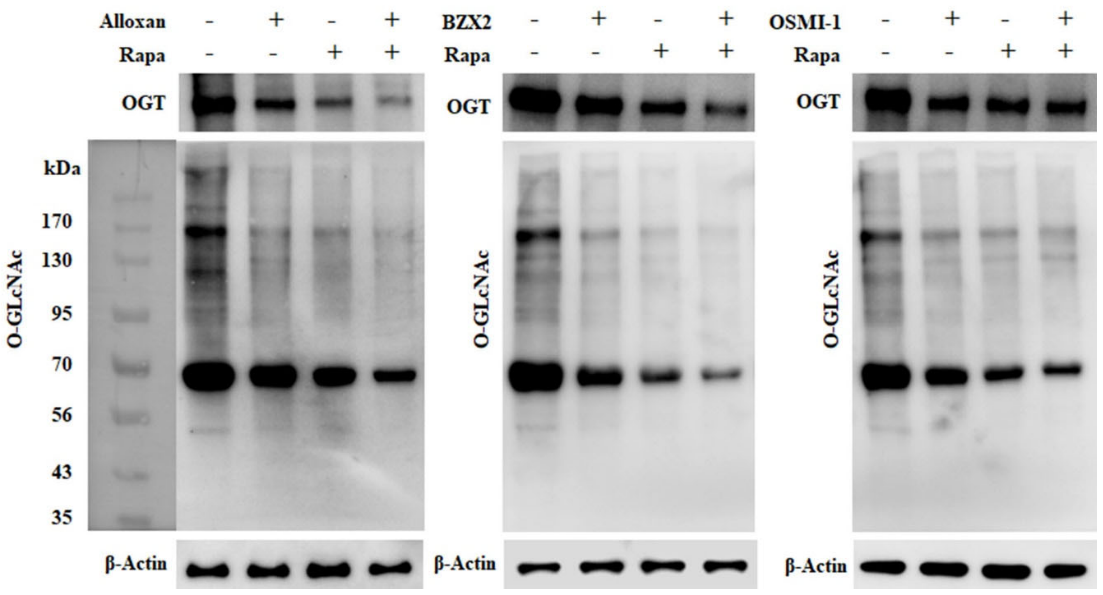

Supplementary Figure 3: Inhibition of OGT regulates mTOR signaling pathway in rat cortical neurons. Neurons were pretreated with rapamycin (100 nM) for 30 min before in the absence/presence of alloxan (5 mM), BZX2 (100 µM), and OSMI-1 (50 µM) for 24 h. OGT and O-GlcNAc were determined by immunoblotting analysis. B-actin was used as a loading control.

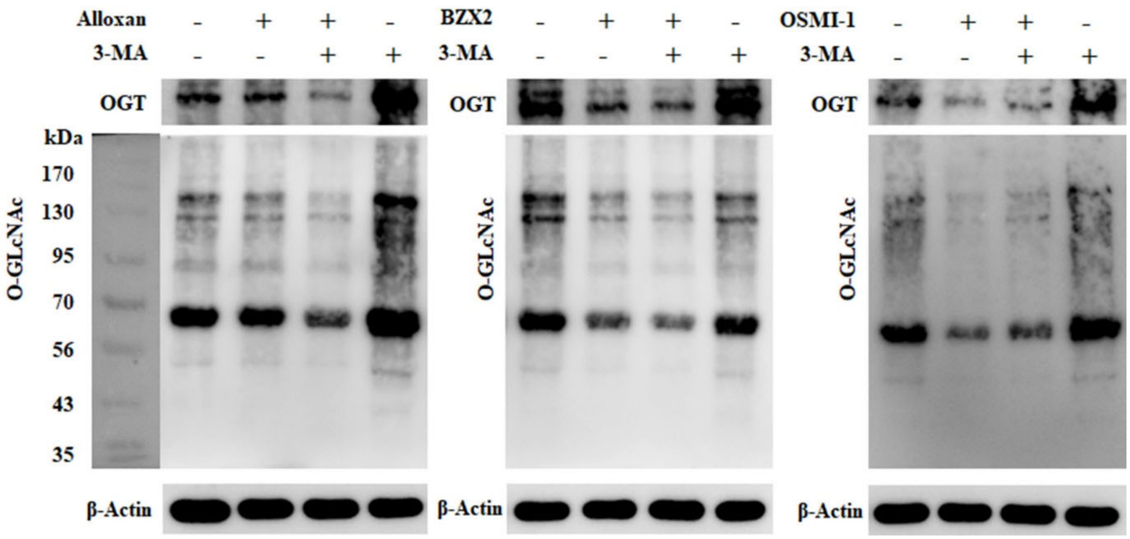

Supplementary Figure 4: Effects of 3-MA on OGT inhibitor-mediated autophagy in rat cortical neurons. Neuronal cells were pre-treated (3 h) with 3-MA (2.5 mM), and subsequently treated with alloxan, BZX2, and OSMI-1 for 24 h. OGT and O-GlcNAc expressions were determined by immunoblot. B-actin was used as a loading control.

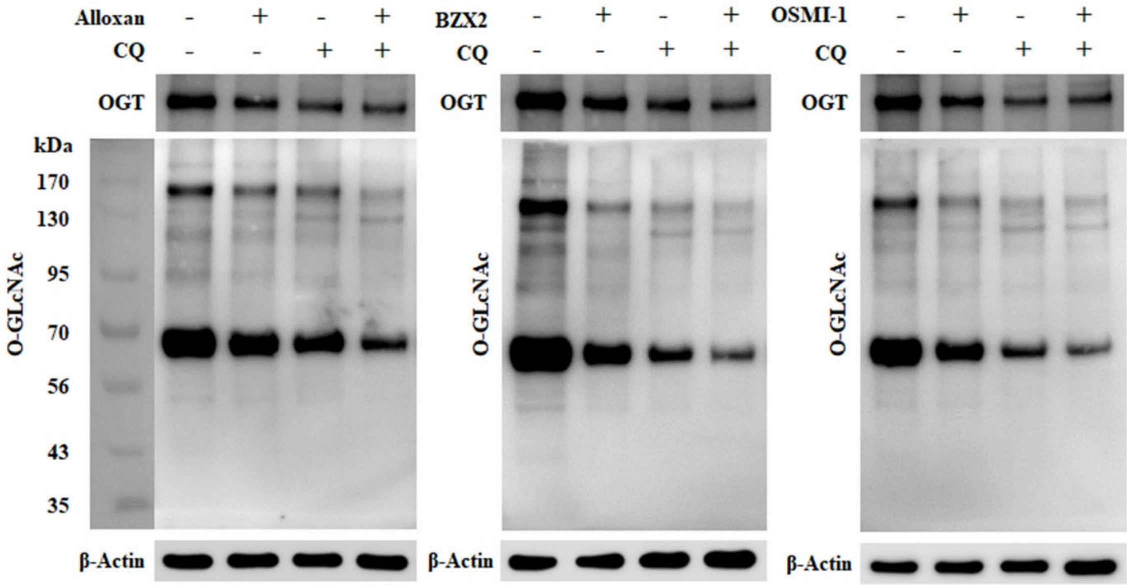

Supplementary Figure 5: Effects of CQ on OGT inhibitor stimulated autophagic activities in rat cortical neurons. Treatment with alloxan, BZX2, and OSMI-1, cortical neurons were incubated with chloroquine (10 μM) for 2 h before harvesting. OGT and O-GlcNAc expression levels were determined by immunoblot analysis. B-actin was used as a loading control.
